# A Case of Spontaneous Atraumatic Subdural Hematoma Without Known Precipitating Factors

**DOI:** 10.7759/cureus.64919

**Published:** 2024-07-19

**Authors:** Sera X Sempson, Timothy L Vo

**Affiliations:** 1 Neurological Surgery, University of Colorado School of Medicine, Aurora, USA; 2 Emergency Medicine, University of Colorado Anschutz Medical Campus, Denver, USA

**Keywords:** pure subdural hematoma, acute spontaneous subdural hematoma, non-traumatic subdural hematoma, subdural hematoma (sdh), acute subdural hematoma

## Abstract

Subdural hematoma (SDH) is a disease commonly seen in both the emergency department and the intensive care unit. Here, we present a case of a woman who developed acute SDH, without any precipitating trauma nor predisposing risk factors. She was managed with hemicraniectomy and SDH evacuation, with subsequent cranioplasty. Routine surveillance imaging found a subsequent, small, and again idiopathic SDH. Comprehensive hematologic workup demonstrated no evidence of coagulopathy. To our knowledge, there are minimal prior case reports published in the literature regarding idiopathic, unprovoked SDH.

## Introduction

A subdural hematoma (SDH) is an abnormal collection of blood located under the dura mater, above the arachnoid layers. The incidence of chronic SDH has increased as the median age of global populations has increased, and estimates range from 1.7 cases per 100,000 persons per year up to 20.6 cases per 100,000 persons per year [1]. Acute SDH is most commonly caused by trauma, however, 3-5% of SDH cases are attributable to vascular anomalies and other precipitating medical conditions [2].

Classically, SDH presents on CT as a hyperdense crescent compressing underlying brain parenchyma. Traumatic SDH is associated with high-energy mechanisms in younger populations, however, in the elderly, they are often seen with lower mechanism forces due to underlying atrophic changes and comorbid disease [3]. Atraumatic SDH is less common, and etiologies include rupture of an arterial-venous malformations, Moyamoya, cancer, aneurysm, coagulopathy, hypertension, and substance abuse [2,4]. Men are more often affected than women, especially in populations under 50 years of age [3]. Chronic SDH affects 1.7-20.6 per 100,000 individuals annually and typically affects the elderly [1,5]. While categories are predicated on time from onset, the actual delineation between acute, subacute, and chronic SDH is less readily apparent, especially when the precipitating insult or event is unknown [6].

## Case presentation

A 44-year-old female presented to the emergency department with a chief complaint of headache. The headache began six days prior to presentation; it was gradual at onset, had worsened over the previous day, and was now severe. There was no history of trauma nor precipitating event, and she did not have a history of primary headache disorder. The headache was bifrontal in nature, was described as a squeezing sensation, and was associated with photophobia. She noted her headache improved with sitting up, worsened with laying down and was unimproved with Tylenol, Ibuprofen, and Aspirin. She denied nausea, vomiting, numbness, tingling or weakness. She was not on oral contraceptives or any other form of hormonal replacement, and she denied a history of tobacco, alcohol, or other drug use. The patient’s medical history was remarkable only for hypothyroidism and family history was noncontributory, including for a history of bleeding tendency. The neurological exam showed no focal deficits. Vital signs were significant for mild hypertension and bradycardia. She was given ketorolac, prochlorperazine, dexamethasone, and fluids with improvement in her headache, and she was discharged.

**Figure 1:**
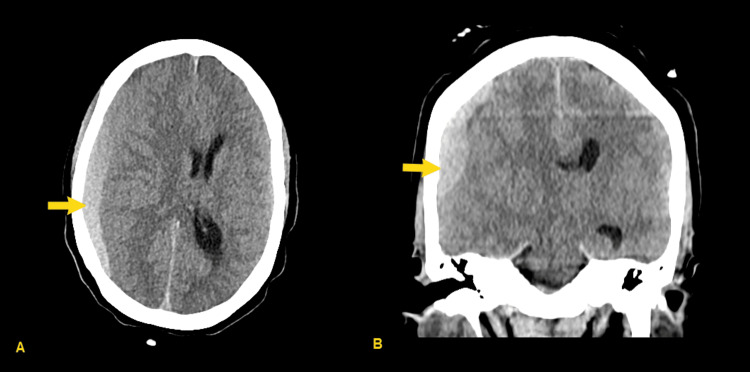
Admission CT head, showing subdural hematoma (SDH) (yellow arrows) with midline shift, subfalcine, and uncal herniation (A) Axial image, (B) Coronal image

**Figure 2:**
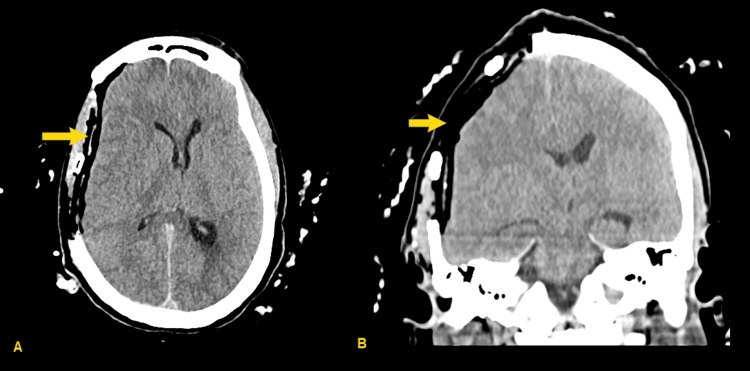
Post-hemicraniectomy CT head, with resolution of previously demonstrated subdural hematoma (SDH) (yellow arrows) (A) Axial image, (B) Coronal image

Four days later, the patient re-presented to the emergency department with continued, worsening headache. At that time, the neurological exam continued to be nonfocal, and her mental status was noted to be normal. Computed tomography (CT) of the head was obtained (Figure 1), revealing acute to subacute subdural hemorrhage with 12 mm midline shift, as well as subfalcine and uncal herniation. CT angiogram (CTA) was performed, showing no intracranial aneurysm or arteriovenous malformation, nor active extravasation into the hematoma. The patient was admitted to the intensive care unit, was given 1 gram of levetiracetam, and underwent hemicraniectomy and subdural evacuation on hospital day 1 (Figure 2); hemicraniectomy was chosen over craniotomy due to her young age, lack of cerebral atrophy, and the size of the lesion. She tolerated the procedure well. She was admitted to the intensive care unit postoperatively for frequent neurological monitoring. She was transferred to a general medical floor bed on postoperative day 3. Her hospital course was uneventful, and she was discharged on hospital day 5.

She returned for admission for planned cranioplasty two months later. This was completed on hospital day 1, and she was admitted to the intensive care unit following the procedure. Her hospital course was uneventful, and she was discharged on hospital day 3.

**Figure 3:**
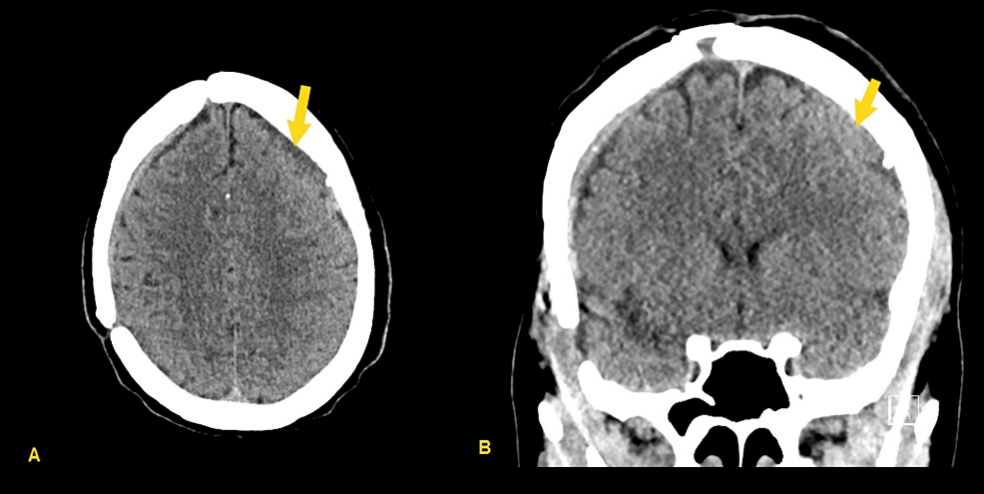
CT head 12 weeks after cranioplasty, showing the development of a new small acute right frontal subdural hematoma (SDH) (yellow arrows), suspected again to be spontaneous (A) Axial image, (B) Coronal image

She continued to follow-up with neurological surgery in clinic, with continued improvement in her functional status. Surveillance interval CT head was performed at eight weeks, showing decreased size of right frontal SDH, however repeat CT head four weeks after that demonstrated new interval development of acute/subacute SDH (Figure 3). She again denied any new trauma at that point, and this new SDH was suspected to be spontaneous as well.

**Figure 4:**
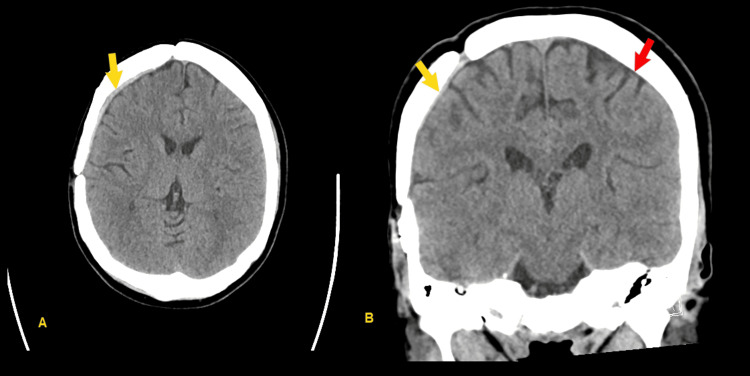
Final CT head, showing near-complete resolution of original subdural hematoma (SDH) (yellow arrows) as well as resolution of second SDH (red arrow) (A) Axial image, (B) Coronal image

Due to small size, the decision was made to manage this second SDH expectantly. Repeat CT head was performed two weeks thereafter, showing stable findings. MRI of the brain with and without contrast was performed to assess for findings of intracranial hypertension or underlying lesion causing recurrent SDH; this showed only mild bilateral smooth dural thickening and enhancement, felt to be most likely postsurgical and related to recent subdural hemorrhage, as well as a 7-mm T2 hyperintense and somewhat T1 hypointense area in the anterior left mesial temporal lobe with facilitated effusion felt most likely to be posttraumatic gliosis. CT head was repeated at six weeks, showing near-complete resolution of SDH (Figure 4).

She was seen by hematology to assess for underlying coagulopathic disorder. Her prothrombin time (PT), partial thromboplastin time (PTT), Factor VIII activity, factor XIII, von Willebrand factor antigen, Ristocetin cofactor, and platelet aggregation and ATP secretion response study were all found to be normal. PFA-100 platelet function analyzer assay was also normal.

## Discussion

Acute spontaneous subdural hemorrhage is rare. A 2014 literature review found only 22 cases published in the literature in patients under the age of 40 [7]. Previously published case series and case reports list bleeding from cortical branches of the middle cerebral artery (MCA) and methamphetamine use as other etiologies of spontaneous SDH [2,8]. Idiopathic spontaneous SDH is even more rare, and the literature here is limited to sporadic case reports [9,10].

The most common presenting symptom of SDH is headache, and other symptoms may also include weakness, balance and gait dysfunction, memory problems, seizure, or even coma [5,11]. For acute SDH, which is oftentimes traumatic, there may be distracting symptoms and other significant co-existing injury. In the case of subacute to chronic SDH, blood accumulation is slower, but symptoms are similar. As a result of chronicity, there is also an inflammatory reaction that encapsulates the bleed in a membrane. In sequela are the development of neocapillaries, enzymatic fibrinolysis, and liquefaction of hematomas [6].

Pathologies that increase intracranial pressure (ICP), including excess cerebrospinal fluid or a mass, oftentimes result in a positional headache, as seen with this patient [12]. A study of chronic SDH in 2018 found that headache is most often secondary to midline shift versus increased intracranial pressure or hematoma pressure [11]. Pain is thus secondary to meningeal stretch. Therefore, unlike the classic orthostatic postural-dependent headache of elevated intracranial pressure, SDH may present with non-postural headaches. For this patient, positional headache was the only clue to a more sinister neurologic pathology.

Neoplasms, infection, and autoimmune disorders are common mimics of SDH, given the overall nonspecific nature of presenting symptoms [13]. Unfortunately, given practicality and the pragmatism of clinical judgement, not all patients with headache receive head CT, and not all pathologic headaches demonstrate classic red-flag features.

The greatest prognostic factor of traumatic SDH is the severity of the primary traumatic insult, more so than the subsequent cerebral damage resulting from the mass effect [3]. Yet still, the ideal response to subdural hematoma involves timely identification, seizure prophylaxis, measures of reducing ICP such as elevating the head of the bed, and bleed reduction with reversal of coagulopathy and ligation of any sites of active bleeding [8].

Definitive treatment of subdural hematoma is monitoring versus surgical intervention. Monitoring is preferred for smaller bleeds that exert a minimal mass effect. For larger, acute subdural hematomas, surgical management includes craniotomy versus decompressive craniectomy [14,15]. While decompressive craniectomy removes a portion of the skull, relieving elevated intracranial pressure, craniotomy employs a temporary flap, evacuating the hematoma with suction and irrigation before refixing the flap into place [16]. A randomized control trial from 2023 and a recent meta-analysis from 2024 comparing the two approaches in traumatic SDH demonstrated similar disability and quality of life outcomes [14,15]. In cases of chronic subdural hematomas that develop over a few days or weeks, burr holes are preferred [1]. In this procedure, small holes are drilled into the skull, allowing for the placement and fixation of a drainage catheter. All approaches run the potential risk of worsening the bleed. Further, treatment extends into the post-operative period, especially for patients with etiologies that predispose to recurrence. For example, reversing underlying coagulopathic states, utilizing tranexamic acid, or meningeal artery embolization may be considered [17]. Debate exists surrounding the level of aggression in approach [18].

## Conclusions

Acute spontaneous subdural hemorrhage is a rare entity, and idiopathic spontaneous subdural hemorrhage is even more so. This case underscores the clinical challenge of diagnosing acute spontaneous subdural hemorrhage, particularly when it occurs without an identifiable precipitating cause. A high index of suspicion for subdural hemorrhage must be maintained in patients presenting with severe headaches, even in the absence of trauma or known risk factors. Further research and case studies are warranted to better understand the pathophysiology and epidemiology of idiopathic spontaneous subdural hemorrhage, as well as to refine diagnostic and prevention strategies for this rare condition.
